# Dental Erosion Prevalence and Its Association With Obesity Among Children With and Without Special Healthcare Needs

**DOI:** 10.3290/j.ohpd.b2259007

**Published:** 2021-11-05

**Authors:** Roshan Noor Mohamed, Sakeenabi Basha, Yousef Al-Thomali, Fatma Salem AlZahrani, Amal Adnan Ashour, Nada Eid Almutair

**Affiliations:** a Assistant Professor, Department of Pedodontics, Faculty of Dentistry, Taif University, Saudi Arabia. Contributed to conception and design; contributed to acquisition, analysis, and interpretation; drafted manuscript; critically revised manuscript; gave final approval; agrees to be accountable for all aspects of work ensuring integrity and accuracy.; b Assistant Professor, Department of Community Dentistry, Faculty of Dentistry, Taif University, Taif, Saudi Arabia. Contributed to conception and design, analysis, and interpretation; drafted manuscript; critically revised manuscript; agrees to be accountable for all aspects of work ensuring integrity and accuracy.; c Associate Professor, Department of Orthodontics, Faculty of Dentistry, Taif University, Taif, Saudi Arabia. Contributed to conception and design; contributed to acquisition, analysis, and interpretation; drafted manuscript; critically revised manuscript; agrees to be accountable for all aspects of work ensuring integrity and accuracy.; d Assistant Professor, Department of Pedodontics, Faculty of Dentistry, Taif University, Taif, Saudi Arabia. Contributed to conception and design; contributed to acquisition, analysis, and interpretation; drafted manuscript; critically revised manuscript; agrees to be accountable for all aspects of work ensuring integrity and accuracy.; e Associate Professor, Department of Oral Pathology, Faculty of Dentistry, Taif University, Taif, Saudi Arabia. Contributed to conception and design; contributed to acquisition, analysis, and interpretation; drafted manuscript; critically revised manuscript; agrees to be accountable for all aspects of work ensuring integrity and accuracy.; f Community Services Coordinator, Department of Community Dentistry, Faculty of Dentistry, Taif University, Taif, Saudi Arabia. Contributed to conception and design; contributed to acquisition, analysis, and interpretation; drafted manuscript; critically revised manuscript; agrees to be accountable for all aspects of work ensuring integrity and accuracy.

**Keywords:** dental erosion, obesity, prevalence, special needs

## Abstract

**Purpose:**

Dental erosion and childhood obesity are associated with a common risk factor, soft drink consumption. The present study aims to assess the prevalence of dental erosion and its association with obesity among children with and without special healthcare needs.

**Materials and Methods:**

A cross-sectional study was conducted on 1,200 school children (400 children with special needs (CSHN) and 800 children without special needs) in the age group of 6 to 16 years. Dental erosion was diagnosed according to World Health Organization (WHO) criteria. Body mass index (BMI; weight/height in kg/m^2^) was recorded for each child. The association of dental erosion and obesity was analysed using multivariate logistic regression analysis.

**Results:**

A total of 331 (27.6%) children presented with dental erosion. Dental erosion prevalence among CSHN was 33.5% and among children without special needs was 24.6%. In the final fully adjusted model, children with obesity presented 2.32 times (95%CI 1.17–4.89, P = 0.001) higher odds ratio (OR) of having dental erosion than normal-weight children. Children who consumed soft drinks ≥ 1 time/day and 2–6 times a week presented with an OR of 2.65 (95%CI 1.23–5.21, P = 0.001) times of dental erosion. Children with chronic vomiting and bulimia presented with a 3.27 (95%CI 1.72–6.12, P = 0.001) times higher OR of dental erosion. Children with gastric reflux presented with a 3.21 (95%CI 1.52 – 5.86, P = 0.001) times higher OR of dental erosion.

**Conclusion:**

The prevalence of dental erosion was slightly higher for special needs children compared to children without special needs. The study outcome suggests that obesity, chronic vomiting, consumption of soft drinks, and gastric reflux are statistically significantly associated with dental erosion.

Dental erosion refers to the irreversible loss of tooth structure by a chemical process without bacterial involvement.^28^ It is a multifactorial disease associated with intrinsic and extrinsic factors that involve oesophageal gastric reflux and or consumption of food and beverages which are acidic.^9, 15, 16, 18^ The prevalence of dental erosion varies from 8.9% to 46% among normal children and adolescents depending upon the method of examination and diagnostic factors, the geographic area involved, age of the participants, the sample selection, and associated risk factors.^17, 27, 28, 31^ The systematic review showed a prevalence of 30.4% (95%CI 23.8–37.0) dental erosion among 8–19-year-old children and adolescents.^24^ Few point prevalence studies conducted in Saudi Arabia showed 8.2% to 26% dental erosion prevalence among school children.^5, 14, 30^ Special needs children are more prone to oral diseases like dental caries and periodontal infections due to associated mental, behavioural or physical conditions limiting their day to day life activities.^10, 21^ Along with these, previous researchers have reported a prevalence of 28% to 48% for dental erosion among special children with cerebral palsy and Down’s syndrome due to associated gastric reflux and vomiting.^1, 7, 8, 13, 29^

Childhood obesity is considered one of the major public health challenges of the twenty-first century due to its associated comorbidities like diabetes mellitus, cardiovascular diseases, and psychological disturbances at a younger age.^11^ Along with general health risks, obesity also increases the risk of oral diseases like dental caries, and periodontal diseases due to high sugar, energy-dense, and low nutrient diet.^20, 23^ As per World Health Organization (WHO) global data, the obesity prevalence has increased consistently among children and adolescents (5–19-year-old age group) over the years from 4% in 1975 to 18% in 2016.^11^ The childhood obesity among Saudi Arabian children has increased from 12.7% in 2006 to 18.2% in 2015.^4^ The carbonated soft drinks consumption among school children and adolescents is found to be popular and frequent in Saudi Arabia.^6^ Soft drink consumption is a common risk factor associated with dental erosion and obesity.^3, 9, 19^ Special needs children are more prone to obesity due to limited physical activity and altered dietary patterns, which may also create oral conditions favourable for dental erosion.^7, 22, 24^ The present study assessed the prevalence of dental erosion and its association with obesity among children with and without special healthcare needs.

## Materials and Methods

### Study Population and Sampling Methods

A cross-sectional study was conducted among 6- to 16-year-old school children, Taif City, Saudi Arabia, from September 2018 to March 2019. The sample size was estimated based on a pilot study conducted among 25 special needs children. With an anticipated population proportion of 40%, power of study 80%, an alpha error of 0.5, a sample size of 375 was estimated, which was rounded to 400 special needs children to counter the non-response bias. Eight hundred children without special needs were selected from the same schools. Thirty schools from five zones of the city were selected by random lottery numbers (five to seven schools in each zone) with both special needs children and children without special needs. From each school, 7–20 special needs children and 14–40 children without special needs were selected by probability proportional to the size random sampling technique. The study was approved (ethical clearance number: 39-11007-0029) by the institutional review board. The parents/guardians of the included study participants consented to the study through written informed consent.

### Questionnaire

The parents/guardians of the children received a pretested semi-structured questionnaire. The face validity of the questionnaire was carried out by one of the authors. To check the internal consistency, the questionnaire was distributed to 25 parents/guardians not included in the study. Cronbach alpha test was used to check internal consistency and was found to be 0.852. The questionnaire included information on the following details:

- Sociodemographic details (age in years, gender, family income, parents’ education level, occupation of parents).
- Dietary habits (frequency and time of consumption of citrus fruits, citrus fruit juices, carbonated drinks, iced tea, squash, sparkling water, lemonade, diet soft drinks, fruit punch).
- General health (frequency of vomiting, gastric reflux or heartburn, nausea, bulimia or anorexia nervosa, medication details).
- Oral hygiene practices (method of teeth cleaning, use of fluoridated or non-fluoridated toothpaste, and frequency of teeth cleaning).
- Vitamin C supplements (frequency, duration)

The disability details of special needs children were taken from school records and categorised into six groups as follows: deafness or blindness or both (DB); Down’s syndrome (DS); autistic disorder (AD); cerebral palsy (CP); intellectual disability (ID); children with multiple disabilities (MD).

### Anthropometric Measurements

Body mass index (BMI: weight/height in kg/m^2^) was calculated for each study participant. Height measurement was done using a stadiometer and the weight (to nearest 100 g) of the children was recorded with light clothing in an upright position. The children were categorised based on their age and gender into four specified groups according to Al-Herbish et al^2^ as follows: underweight – less than 5th percentile; normal weight – 5th percentile to less than 84th percentile; overweight – 85th to less than 95th percentile; and obese – equal to or greater than the 95th percentile.

### Oral Examination

A single examiner examined all the children under natural daylight using a sterile community periodontal index (CPI) probe and plane mouth mirror. The dental erosion was diagnosed according to WHO criteria.^33^ The number of teeth involved was recorded by scoring all the teeth with the recording of the highest score if erosion differs on different tooth surfaces: Score 0 = No signs of erosion, Score 1 = Enamel lesion, Score 2 = Dentinal lesions, Score 3 = Pulp involvement. Teeth with gross dental caries, crown placement, and developmental defects was excluded from the examination. The examiner was trained using photos of varying levels of dental erosion. Intraexaminer calibration was determined by examining 25 pre-selected children to represent varying levels of dental erosion. The children were examined at two different times, with an interval of one day between the secessions (Kappa value of 0.92, P <0.05).

### Statistical Analysis

Descriptive summary statistics were determined for all independent and outcome variables of dental erosion. The Chi-square test followed by pair-wise Z test and Bonferroni correction was used for the testing difference in proportion. Multivariable adjusted logistic regression with forward stepwise analysis was used at first to determine the relationships between dental erosion (yes [scores 1, 2, 3 grouped]/no [with score 0]), and all included independent variables. In the next step, the final fully adjusted model was generated by using the covariates with P ≤ 0.05. The Statistical Package for Social Science version 24 (IBM SPSS Statistics, IBM, Armonk, NY) was used. All the statistical tests were two-sided with the statistical significance level P <0.05.

## Results

The mean BMI for the whole study population was 24.32 (± 3.26). The mean BMI for CSHN was 25.17 (± 2.93) and for children without special needs was 24.32 (±2.67). One hundred and twenty-three (30.8%) children presented with intellectual disability, 107 (26.8%) children with autism, 70 (17.5%) children with Down’s syndrome, 43 (10.8%) with CP, 33 (8.2%) with deafness or blindness or both, and 24 (6%) with multiple disabilities. Table 1 presents BMI categories according to age, gender, and soft drink consumption. Obesity prevalence was slightly higher among children who consumed soft drinks, but the difference was not statistically significant. Total of 331 (27.6%) children presented with dental erosion. Dental erosion prevalence among CSHN was 33.5% and among children without special needs was 24.6%. Table 2 presents the prevalence of dental erosion according to the variables studied among children with and without special needs. Obese and overweight children presented a higher prevalence of dental erosion than normal-weight children (P = 0.042). Children who consumed soft drinks ≥ 1 times/day had a higher prevalence of dental erosion than those who never consumed soft drinks (P = 0.034). Children with chronic vomiting and gastric reflux presented with a higher prevalence of dental erosion (P = 0.001). Dental erosion was detected among 58.1% of children with CP (P = 0.034).

**Table 1:** Body mass index categories according to age, gender, and soft drink consumption

Variable	BMI categories			
	Underweight n (%)	Normal weight n (%)	Overweight n (%)	Obese n (%)
**CSHN**				
**Age in years**				
6–11 years (n = 160)	9 (5.6)^a^	76 (47.5)^a^	40 (25)^a^	35 (21.9)^a^
12–16 years (n = 240)	14 (5.8)^a^	115 (47.9)^a^	37 (15.5)^a^	74 (30.8)^a^
Chi-square test, P value	0.121			
**Gender**				
Boys (n = 180)	13 (7.2)^a^	86( 47.8)^a^	33 (18.3)^a^	48 (26.7)^a^
Girls (n = 220)	10 (4.6)^a^	105 (47.7)^a^	44 (20)^a^	61 (27.7)^a^
Chi-square test, P value	0.092			
**Consumption of soft drinks**				
Yes (n = 274)	10 (3.6)^a^	134 (48.9)^a^	49 (17.9)^a^	81 (29.6)^a^
No (n = 126)	13 (10.3)^a^	57 (45.2)^a^	28 (22.2)^a^	28 (22.2)^a^
Chi-square test, P value	0.063			
**CWSHN**				
**Age in years**				
6–11 years (n = 340)	12 (3.6)^a^	198 (58.2)^a^	77 (22.6)^a^	53(15.6)^a^
12–16 years (n = 460)	10 (2.2)^a^	230 (50)^a^	85 (18.5)^a^	135 (29.3)^a^
Chi-square test, P value	0.084			
**Gender**				
Boys (n = 320 )	9 (2.8)^a^	183 (57.2)^a^	72 (22.5)^a^	56 (17.5)^a^
Girls (n = 480)	13 (2.7)^a^	245 (51)^a^	90 (18.8)^a^	132 (27.5)^a^
Chi-square test, P value	0.093			
**Consumption of soft drinks**				
Yes (n = 680)	8 (1.2)^a^	364 (53.5)^a^	145 (21.3)^a^	163 (24)^a^
No (n = 120)	14 (11.7)^a^	64 (53.3)^a^	17 (14.2)^a^	25 (20.8)^a^
Chi-square test, P value	0.052			

CSHN, children with special health needs; CWSHN, children without special health needs; a, pair-wise Z test with Bonferroni correction, P >0.05.

**Table 2:** Dental erosion prevalence according to variables among children with and without special healthcare needs

Variables		CSHN	DE present	CWSHN	DE present	Chi-square test, P value
		n	n (%)	n	n (%)	
Age in years	6–11 years	160	38 (23.8)^a^	340	54 (15.9)^a^	0.031
	12–16 years	240	96 (40)^b^	460	143 (31.1)^a^	
Gender	Male	180	56 (31.1)^a^	320	66 (20.6)^a^	0.062
	Female	220	78 (35.5)^a^	480	131 (27.3)^a^	
BMI	Underweight	23	4 (17.4)^a^	22	3 (13.6)^a^	0.042
	Normal weight	191	57 (29.8)^a^	428	86 (20.1)^a, b^	
	Overweight	77	21 (27.3)^b^	162	28 (17.3)^a^	
	Obese	109	52 (47.7)^b^	188	80 (42.6)^b^	
Consumption of soft drinks	≤ once a week	87	37 (42.5)^b^	235	51 (21.7)^a^	0.034
	2–6 days a week	114	43 (37.7)^a, b^	324	74 (22.8)^a, b^	
	≥ once a day	73	29 (39.7)^b^	121	53 (43.8)^b^	
	Never	126	25 (19.8)^a^	120	19 (15.8)^a^	
Citrus fruit consumption	Yes	231	95 (41.1)^a^	568	145 (25.5)^a^	0.063
	No	169	39 (23.1)^a^	232	52 (22.4)^a^	
Chronic vomiting or bulimia	Yes	17	12 (70.6)^c^	27	23 (85.2)^c^	0.001
	No	383	122 (31.9)^a^	773	174 (22.5)^a^	
Consumption of vitamin C	Yes	98	25 (25.5)^a^	108	32 (29.6)^a^	0.092
	No	302	109 (36.1)^a^	692	165 (23.8)^a^	
Gastric reflux	Yes	83	57 (68.7)^c^	39	22 (56.4)^c^	0.001
	No	317	77 (24.3)^a^	761	175 (23)^a^	
Type of disability	DS	70	21 (30)^a^	NA	NA	NA
	AD	107	16 (15)^a^	NA	NA	
	ID	123	55 (44.7)^a^	NA	NA	
	CP	43	25 (58.1)^b^	NA	NA	
	DB	33	10 (30.3)^a^	NA	NA	
	MD	24	7 (29.2)^a^	NA	NA	
	Chi-square test, P value	0.034				

CSHN, children with special health needs; CWSHN, children without special health needs; DE, dental erosion, DS, Down’s syndrome; AD, autistic disorder; ID, intellectual disability; CP, cerebral palsy; MD, children with multiple disabilities; NA, not applicable; ^a, a and b^ Pair-wise Z test with Bonferroni correction, P > 0.05; ^b, c^ Pair-wise Z test with Bonferroni correction, P< 0.05.

Dental erosion was detected in 961 permanent teeth and 409 deciduous teeth. Permanent mandibular incisors and deciduous maxillary molars were the most frequently affected teeth. Enamel erosion was the most frequent type detected (Table 3).

**Table 3:** Dental erosion category according to teeth affected

Teeth affected	Dental erosion categories			
	1	2	3	Total
Upper anterior, permanent (11, 21, 12, 22, 13, 23) (n = 5,720)	123	72	21	216
Lower anterior, permanent (31, 32, 41, 42, 13, 23) (6, 110)	211	131	56	398
Upper posterior, permanent (14, 15, 16, 17, 24, 25, 26, 27) (n = 4,920)	92	47	4	143
Lower posterior, permanent (34, 35, 36, 37, 44, 45, 46, 47) (n = 5,112)	107	82	15	204
Upper anterior, deciduous (51, 52, 61, 62, 53, 63) (n = 450)	41	28	10	79
Lower anterior, deciduous (71, 72, 81, 82, 73, 83) (n = 416)	48	21	11	80
Upper posterior, deciduous (54, 55, 64, 65) (n = 2,980)	39	71	18	128
Lower posterior, deciduous (74, 75, 84, 85) (n = 2,854)	61	48	13	122
Total	722	500	148	1,370

Table 4 presents the initial adjusted regression analysis between dental erosion and all included independent variables among children with and without special needs. The obesity, soft drinks consumption ≥ 1 time/day and 2–6 times a week, chronic vomiting and bulimia, and the presence of gastric reflux were statistically significantly associated with dental erosion prevalence (P <0.05).

**Table 4:** Adjusted odds ratio (OR) for dental erosion occurrence in CSHN and CWSHN

Variables	CSHN		CWSHN	
	Adjusted OR (95% CI)	P value	Adjusted OR (95% CI)	P value
**Age in years**				
6–11 years	1		1	
12–16 years	0.98 (0.62–1.87)	0.092	0.87 (0.54–1.76)	0.147
Gender				
Male	1		1	
Female	0.79 (0.32–1.36)	0.124	0.65 (0.22–1.08)	0.113
**BMI**				
Underweight and NW	1		1	
Obese and overweight	2.13 (0.98–4.12)	0.001	1.97 (0.45–3.21)	0.032
**Consumption of soft drinks**				
Never and ≤ once a week	1		1	
2–6 days a week and ≥ once a day	2.41 (1.02–5.36)	0.001	2.16 (0.98–4.87)	0.001
**Citrus fruit consumption**				
Yes	0.83 (0.11–1.62)	0.128	0.74 (0.12–1.32)	0.116
No	1		1	
**Chronic vomiting or bulimia**				
Yes	2.89 (1.06–5.47)	0.001	2.23 (0.97–4.32)	0.001
No	1		1	
**Consumption of vitamin C**				
Yes	0.98 (0.13–1.25)	0.093	0.84 (0.17–1.11)	0.142
No	1		1	
**Gastric reflux**				
Yes	2.96 (1.13–5.32)	0.001	2.11 (0.78–4.21)	0.001
No	1		1	
**Type of disability**				
AD	1		NA	NA
DS	0.98 (0.12–1.43)	0.092	NA	NA
ID	1.03 (0.17–1.93)	0.074	NA	NA
CP	1.47 (0.23–2.86)	0.062	NA	NA
DB	0.96 (0.11–1.59)	0.121	NA	NA
MD	0.97 (0.13–1.82)	0.094	NA	NA

CSHN, children with special health needs; CWSHN, children without special health needs; NW, normal weight; DS, Down’s syndrome; AD, autistic disorder; ID, intellectual disability, CP, cerebral palsy; MD, children with multiple disabilities; NA, not applicable; 1, reference value; OR, odds ratio; CI, confidence interval.

Table 5 presents final fully adjusted model for association between dental erosion and associated factors. The odds ratio (OR) for dental erosion was 2.32 (95%CI 1.17–4.89, P = 0.001) for obese children, 2.65 (95%CI 1.23–5.21, P = 0.001) for children who consumed soft drinks ≥ 1 time/day and 2–6 times a week, 3.27 (95%CI 1.72–6.12, P = 0.001) for children with chronic vomiting and bulimia, and 3.21 (95%CI 1.52–5.86, P = 0.001) for children with gastric reflux.

**Table 5:** Final multivariate fully adjusted model for association between dental erosion and the associated factors

Variables	Adjusted OR (95% CI)	P value
**BMI**		
Underweight and normal weight	1	
Obese and overweight	2.32 (1.17–4.89)	0.001
**Consumption of soft drinks**		
Never and ≤ once a week	1	
2–6 days a week and ≥ once a day	2.65 (1.23–5.21)	0.001
**Chronic vomiting or bulimia**		
Yes	3.27 (1.72–6.12)	0.001
No	1	
**Gastric reflux**		
Yes	3.21 (1.52–5.86)	0.001
No	1	

Wald test Chi-square: P ≤ 0.001, 1, Reference value; OR, odds ratio; CI, confidence interval.

## Discussion

Dental erosion and obesity are multifactorial diseases with soft drink consumption as a common risk factor between the two. The present study was the first to assess the prevalence and association between dental erosion and obesity among CSHN compared to children without special needs. Dental erosion prevalence among CSHN was 33.5% and among children without special needs was 24.6%. Published research has shown a wide range of dental erosion prevalence (8.9% to 46%) among normal children,^17, 25, 27, 28, 31^ and 28% to 48% among children with special needs^1, 7, 8, 13, 29^ depending upon the method of examination, geographic area involved, sample selection, and associated risk factors. Few point prevalence studies conducted in Saudi Arabia showed 8.2% to 26% dental erosion prevalence among normal school children,^5, 14, 30^ and 36% among special needs children.^7^ The present study result showed a higher prevalence of dental erosion among 12–16-year-old than 6–11-year-old children. The exfoliation of primary teeth and lesser time of exposure of newly erupted permanent teeth to erosive risk factors may be attributed to a lower prevalence of dental erosion in younger children.^7, 27, 28^

The children with obesity presented with 2.32 times higher OR of dental erosion than normal children. This may be due to a common risk factor associated with dental erosion and obesity that is the consumption of soft drinks and gastric reflux. Soft drinks with high sugar content and low pH increase the risk of obesity and dental erosion, respectively.^9, 19^ Obesity, in turn, causes gastric reflux,^12, 34^ which leads to dental erosion.^7, 18^ In agreementwith the results of the present study, the study by Salas et al^26^ showed that private school children with obesity presented with odds of 3.26 (95%CI 1.38–7.69) times of dental erosion prevalence. However, no statistically significant association was reported for obese public school children. The high consumption of soft drinks among private school children contributed to a statistically significant association with dental erosion prevalence and obesity. In contrast to the present observation, the study by Basha et al^7^ reported no association between dental erosion and obesity among special needs children irrespective of high soft drinks consumption. The reason may be due to the low sample size that the statistical power might have not been sufficient to detect the differences.

The term ‘soft drink’ usually refers to flavoured, carbonated, non-alcoholic beverages with caloric or non-caloric sweeteners. Although both sugary and non-sugary soft drinks can induce dental erosion, obesity is associated with the consumption of sugary soft drinks.^19^ Carbonated soft drinks consumption among school children and adolescents is found to be popular and frequent in Saudi Arabia due to the warm climate and high spending lifestyle.^6^ In the present study, 68.5% of CSHN and 85% of children without special needs consumed soft drinks, and in agreement with previous researches,^9, 15–17, 27, 28, 31^ the present study showed soft drink consumption ≥ 1 time/day is significantly associated with the prevalence of dental erosion. According to the regression analysis, there were 2.65 times higher odds of dental erosion with increased frequency of soft drink consumption. This may be due to low pH and reduced buffering capacity of non-alcoholic acidic beverages, which increased tooth erosion.^9^

The present study showed children with chronic vomiting and bulimia presented with 3.27 times higher OR of dental erosion. In the present study, 20.8% of children with special needs and 4.9% of children without special needs presented with gastric reflux, and the presence of gastric reflux showed 3.21 times higher odds for developing dental erosion. The result is in agreement with previous researches, which showed titratable acidity and lower pH of gastric juice increased dental erosion prevalence.^7, 18^ However, in contrast to the present study result, Wild et al^32^ and Jensdottir et al^15^ reported no association between dental erosion and gastric reflux. The reason may be due to the inclusion of the older age population, and the erosion of permanent teeth by extrinsic acid could have influenced the result in these studies.^15, 32^

The study limitation includes its cross-sectional nature, which limited the causal association between dental erosion and risk factors. The recall bias while recording dietary details cannot be ruled out. The World Health Organization criteria used to diagnose dental erosion can also be misleading, as often the whole of the enamel surface can be impacted without dentine being exposed.

## Conclusion

The present study concluded that special needs children presented with a slightly higher prevalence of dental erosion compared to children without special needs. Dental erosion was statistically significantly associated with obesity, chronic vomiting, consumption of soft drinks, and gastric reflux. Due to the common risk factor associated with dental erosion and obesity, there is a need to adopt a collaborative approach to tackle both dental erosion and childhood obesity with a combined effort by the public and the private sector to manage these two diseases together by controlling the consumption of soft drinks.
